# Co-ingestion of carbohydrate and whey protein isolates enhance PGC-1α mRNA expression: a randomised, single blind, cross over study

**DOI:** 10.1186/1550-2783-10-8

**Published:** 2013-02-12

**Authors:** Karen M Hill, Christos G Stathis, Esther Grinfeld, Alan Hayes, Andrew J McAinch

**Affiliations:** 1 Biomedical and Lifestyle Diseases Unit, College of Health and Biomedicine, Victoria University, PO Box 14428, Melbourne, VIC 8001, Australia; 2 Institute of Sport, Exercise and Active Living, Victoria University, Melbourne, Australia

**Keywords:** Muscle protein synthesis, Protein supplementation, Endurance exercise adaptations, Mitochondrial biogenesis

## Abstract

**Background:**

Whey protein isolates (WPI) supplementation is known to improve resistance training adaptations. However, limited information is available on the effects of WPI plus carbohydrate (CHO) supplementation on endurance training adaptations.

**Method:**

Six endurance trained male cyclists and triathletes (age 29 ± 4 years, weight 74 ± 2 kg, VO_2 max_ 63 ± 3 ml oxygen^.^ kg^-1.^ Min^-1^, height 183 ± 5 cm; mean ± SEM) were randomly assigned to one of two dietary interventions in a single blind cross over design; CHO or CHO + WPI. Each dietary intervention was followed for 16 days which included the last 2 days having increased CHO content, representing a CHO loading phase. The dietary interventions were iso-caloric and carbohydrate content matched. On completion of the dietary intervention, participants performed an exercise bout, consisting of cycling for 60 min at 70% VO_2 max,_ followed by time trial to exhaustion at 90% VO_2 max_ and recovered in the laboratory for 6 hours. Blood samples and muscle biopsies were taken at various time points at rest and through the exercise trial and recovery.

**Results:**

Compared to CHO, CHO + WPI increased plasma insulin during recovery at 180 mins (P < 0.05) and peroxisome proliferator-activated receptor gamma coactivator-1 alpha (PGC-1α) mRNA expression at the end of 6 hours of recovery (P < 0.05). Muscle glycogen did not differ between the two trials.

**Conclusion:**

This study showed co-ingestion of CHO + WPI may have beneficial effects on recovery and adaptations to endurance exercise via, increased insulin response and up regulation of PGC-1α mRNA expression.

## Background

Optimal nutrition is not only required for normal physiological functioning, but the nutritional status of an endurance athlete can negatively or positively impact their sporting performance [1]. Nutritional requirements of endurance athletes include higher energy needs to fuel exercise and replace glycogen stores and increased protein intake to support muscle protein turnover. During endurance exercise major disturbances to cellular homeostasis, substrate stores and utilization occur in the muscle [2]. Recovery from endurance training sessions is fundamental, as the muscle damage caused during exercise partly due to muscle contraction and hormonal changes that result in the breakdown of muscle protein, continues once exercise is ceased [3]. This damage can impair subsequent muscle function, delivery of nutrients, glycogen resynthesis rates and impair protein synthesis pathways [3].

Repeated bouts of endurance exercise result in structural, metabolic and physiological adaptations that enable improved performance [4]. Long term adaptations are a cumulative result of successive training sessions and the post exercise period is critical in allowing these processes [2]. During recovery the activation of several major signalling pathways occurs in the first few hours before returning to baseline within 24 hours [2]. Recovery from endurance exercise requires muscle glycogen stores to be replenished and damaged muscle to be repaired [5].

Nutrition is a key component supporting heavy training and competition [6]. The primary fuel source during endurance events is muscle glycogen [7,8]. It is well documented that depletion of intramuscular glycogen stores can limit performance during prolonged exercise [9]. Maximising pre-exercise glycogen levels through carbohydrate loading has become well practiced by athletes, in addition to refuelling immediately post exercise to optimise muscle glycogen restoration [10]. However, carbohydrates alone are not enough to stimulate significant protein synthesis and the adaptive response to endurance exercise [11].

Protein is an extremely important substrate, due to the influence it exerts over the regulation rates of muscle protein synthesis (MPS) and the subsequent effects on the phenotype of skeletal muscle [12]. Muscle adaptations depend on the availability of sufficient protein [2]. The type of protein consumed can affect the recovery process due to differences in the digestion rate of the protein and concentration of proteins [11]. Micellar casein proteins are released from the stomach slower than whey protein isolates. Therefore, whey produces a faster, transient increase in plasma amino acid concentration and potentially an improved availability of amino acids [13]. Whey protein isolates, compared with other protein sources, are more effective at promoting protein synthesis following resistance exercise due to the high concentration of essential and branched chain amino acids [14].

The mode of exercise influences the subsequent muscle adaptations, with endurance exercise primarily resulting in increased muscle oxidative capacity and resistance exercise predominantly resulting in muscle hypertrophy [15]. Endurance training improves skeletal muscle adaptations by increases in activators of mitochondrial biogenesis such as peroxisome proliferator-activated receptor gamma coactivator-1 alpha (PGC-1α) [16,17].

The regulation of protein synthesis involves several signalling pathways. These are influenced by amino acids, insulin and mechanical stimulation [18]. A large body of research exists which demonstrates the benefits of protein supplementation with resistance exercise [14,19,20]. However, limited research exists on the benefits of protein supplementation for athletes undertaking endurance training. In particular, the effects of co-ingestion of whey protein isolates and carbohydrate on endurance exercise recovery and PGC-1α pathway.

The present study investigates 2 weeks of co-ingestion of whey protein isolates plus ample carbohydrate (CHO + WPI) on endurance performance and recovery, compared to an isocaloric, carbohydrate content matched group (CHO). We hypothesized that CHO + WPI will improve performance and recovery by increasing muscle glycogen levels and facilitating adaptive response, compared to CHO alone.

## Methods

### Subjects

Six healthy endurance trained cyclists and triathletes volunteered to complete the study (age 29 ± 4 years, weight 74 ± 2 kg, VO_2 max_ 63 ± 3 ml oxygen^.^ kg^-1.^ min, height 183 ± 5 cm; mean ± SEM). This study was approved by Victoria University Human Research Ethics Committee. The purpose and potential risks of the experiment were explained to participants prior to them providing written informed consent. Participants completed a standard medical questionnaire prior to commencing trials. Involvement in this study required attainment of a maximal oxygen consumption of at least 60 ml oxygen kg^-1^ min^-1^ and not having consumed whey protein supplements in the 12 weeks prior to the study.

### Preliminary measurements

Participants reported to the laboratory for a VO_2 max_ cycling test on a cycle ergometer. The exercise test consisted of 3 min at 3 sub-maximal workloads followed by subsequent increments of 25 watts (W) every min until fatigue. During the test, subjects’ heart rate (HR) was monitored and respiratory gases collected continuously for gas analysis. Respiratory gas measurements were measured using open circuit spirometry indirect calorimetry using a metabolic cart.

Data obtained from participants VO_2 max_ was used to calculate their workloads (70% and 90% VO_2 max_) for the exercise trial. A standard curve was constructed from the 3 sub-maximal workloads and VO_2_. The predicted VO_2 max_ was then used to calculate the percentage workloads (W) according to the linear equation generated by the standard curve.

On completion of testing, participants were introduced to the dietary regimes and trial procedures used during the study. It was requested that participants maintain their training throughout the dietary interventions and washout period.

### Study design

A randomised, single blind cross over design was used to test the effect of whey protein isolates supplementation on endurance performance and recovery. The dietary interventions were randomly assigned and participants were blinded to the intervention, by matching CHO beverage and CHO + WPI beverage for taste, smell and appearance. Each dietary protocol was followed for a total of 16 d (14 d followed by 2 d CHO loading phase) with a 4 week wash out period to separate the dietary interventions.

Dietary interventions were isocaloric and CHO content matched (see Table 1 for nutritional value of diets). Diets were isocaloric through altering the amount of fat consumed, however the total fat content in the CHO group still contributed less than 30% of total energy. The extra 1.2 g ^.^ kg^-1.^ bw/d of protein was supplemented with whey protein isolates (Table 2) and was provided in a readymade sports drink (Table 3; provided courtesy of MG Nutritionals, Australia). The CHO trial consumed the sports drink, minus the WPI. On training days participants were instructed to consume the drink during and after training sessions and on non-training days to consume any time throughout the day.

**Table 1:** Carbohydrate (CHO), protein (PRO) and fat content of dietary interventions for carbohydrate (CHO) and carbohydrate and whey protein isolates (CHO + WPI)

**14 days**				**2 day CHO loading**		
	**CHO (g**^**. **^**kg**^**-1. **^**bw/day)**	**PRO (g**^**. **^**kg**^**-1. **^**bw/day)**	**Fat (g**^**. **^**kg**^**-1. **^**bw/day)**	**CHO (g**^**. **^**kg**^**-1. **^**bw/day)**	**Pro (g**^**. **^**kg**^**-1. **^**bw/day)**	**Fat (g**^**. **^**kg**^**-1. **^**bw/day)**
CHO	8	1.2	1.7	10	1.2	1.7
CHO + WPI	8	2.4	1.1	10	2.4	1.1

**Table 2:** Amino acid profile of whey protein isolate supplement used in the sports beverages

**Component**	**% w/w**
Alanine	5.2
Arginine	2.7
Aspartic acid	10.6
Cystine	1.9
Glutamic acid	17.5
Glycine	1.3
*Histidine	1.6
* Isoleucine	6.1
* Leucine	15.3
* Lysine	10.4
* Methionine	2.6
* Phenylalanine	3.4
Proline	4.4
Serine	3.2
* Threonine	4.4
* Tryptophan	2.3
Tyrosine	4.1
* Valine	5.2

* indicates essential amino acid.

**Table 3:** Nutritional information for the sports beverage

**Average quantity per 100 ml**	**CHO**	**WPI**
Energy	119 kJ	180 kJ
Protein	0 g	3.6 g
Fat	0 g	0 g
Carbohydrate	7 g	7 g
Sodium	30 mg	30 mg
Potassium	40 mg	40 mg

Participants were provided with all their meals and snacks throughout the duration of the dietary interventions to ensure consistency in energy and macronutrient levels and to assist with compliance. Additionally, participants were provided with check-off sheets to facilitate documenting food intake.

### Experimental trials

After completing the 16 d dietary intervention (CHO or CHO + WPI), participants reported to the laboratory after an overnight fast. The exercise trial was completed on a cycle ergometer which consisted of cycling for 60 min at 70% VO_2 max_ followed by 2 min break, then cycling to fatigue at 90% VO_2 max_. Following this, subjects recovered in the laboratory for 6 h.

During the 6 h recovery period participants followed the dietary intervention they had been on prior to their exercise trial (CHO or CHO + WPI). If they were consuming the CHO diet, they consumed 4 g ^.^ kg^-1.^ bw carbohydrate, 0.6 g ^.^ kg^-1.^ bw fat and 0.4 g ^.^ kg^-1.^ bw protein. Following the CHO + WPI diet participants consumed 4 g ^.^ kg^-1.^ bw carbohydrate, 0.4 g ^.^ kg^-1.^ bw fat and 1.1 g ^.^ kg^-1.^ bw protein during the first 3 h of the 6 h recovery period. The protein source during recovery for the CHO + WPI group was predominantly whey protein isolate provided in the sports drinks (0.7 g ^.^ kg^-1.^ bw). Recovery nutrition was carbohydrate matched and isocaloric by altering the fat content in the breakfast provided.

Venous blood samples were taken from an antecubital vein at rest, every 20 min during cycling at 70% VO_2_ _max_, and on completion of cycling at 90% VO_2_ _max_. Blood was taken every 10 min during the first hour and every hour after this for the remaining 6 h of recovery. Plasma was subsequently analysed for glucose and insulin concentration.

Muscle biopsies were taken at rest, the end of 60 min cycling at 70% VO_2_ _max_ during the 2 min break, on completion of cycling to fatigue at 90% VO_2_ _max_ and the end of 6 h recovery. Muscle biopsies were obtained from the vastus lateralis. Leg selection was random and in the second trial the contra lateral leg was biopsied. The biopsy site was prepared under local anaesthesia (1% xylocaine) and an incision was made at the site in the skin (one incision per sample) prior to exercise. Muscle samples were taken using the Bergstrom [21] procedure as modified for suction [22]. Muscle samples were frozen in liquid nitrogen for subsequent analysis.

One portion of frozen muscle was used to analyse muscle glycogen. Muscle samples were freeze dried and powdered and any obvious blood and connective tissue removed. The samples were weighed and tissue extracted in acid and neutralized in preparation for determination of muscle glycogen. Muscle glycogen was measured using an enzymatic assay adapted for fluorometry [23].

Messenger RNA (mRNA) expression of glycogen synthase, PGC-1α and adenosine monophosphate-activated protein kinase-alpha 2 (AMPK-α2) was analyzed by ‘real-time’ PCR. ‘Real–time’ PCR was conducted using MyiQ™ single colour ‘real-time’ PCR detection system (Bio-Rad Laboratories, Hercules, CA) with iQ™ SYBR Green Supermix (Bio-Rad Laboratories, Hercules, CA) as the fluorescent agent. Forward and reverse oligonucleotide primers for the genes of interest were designed using OligoPerfect™ Suite (Invitrogen, Melbourne, Australia) with sequences obtained from GenBank. Selective gene homology was confirmed with BLAST. To compensate for variations in RNA input amounts and to reverse transcriptase efficiency mRNA abundance of housekeeping genes, GAPDH and cyclophilin was quantified and the expression of the genes of interest was normalised to this (Forward and reverse oligonucleotide primers are shown in Table 4). ‘Real–time’ PCR reactions (total volume 20 μl) were primed with 2.5 ng of cDNA and were run for 40 or 50 cycles of 95°C for 15 sec and 60°C for 60 sec. Relative changes in mRNA abundance was quantified using the 2^-ΔΔCT^ method as previously detailed [24] and reported in arbitrary units.

**Table 4:** Oligonucleotide primers for ‘Real – Time’ PCR primers

**Human genes**	**Accession number**	**Forward primer**	**Reverse primer**
		**(5′ - 3′)**	**(5′ - 3′)**
**Cyclophilin**	NM_021130.3	CATCTGCACTGCCAAGACTGA	TTCATGCCTTCTTTCACTTTGC
**GAPDH**	NM_002046.3	CAACGACCACTTTGTCAAGC	TTACTCCTTGGAGGCCATGT
**AMPK-α2**	NM_006252.3	AACTGCAGAGAGCCATTCACTTT	GGTGAAACTGAAGACAATGTGCTT
**PGC-1α**	NM_013261.3	CAAGCCAAACCAACAACTTTATCTCT	CACACTTAAGGTGCGTTCAATAGTC
**Glycogen synthase**	NM_002103.4	GCTCCCTGTGGACTATGAGG	ATTCCCATAACCGTGCACTC

### Statistical analysis

All data is expressed as means ± standard error of the mean (SEM). Two way repeated measures ANOVA (treatment × time) was used to compare means, using GraphPad Prism (version 5.01, GraphPad Software Inc., San Diego, CA, USA). Significance was set at P < 0.05. When significance was detected Bonferoni post hoc test was used to determine where significance occurred.

## Results

**Figure 1:**
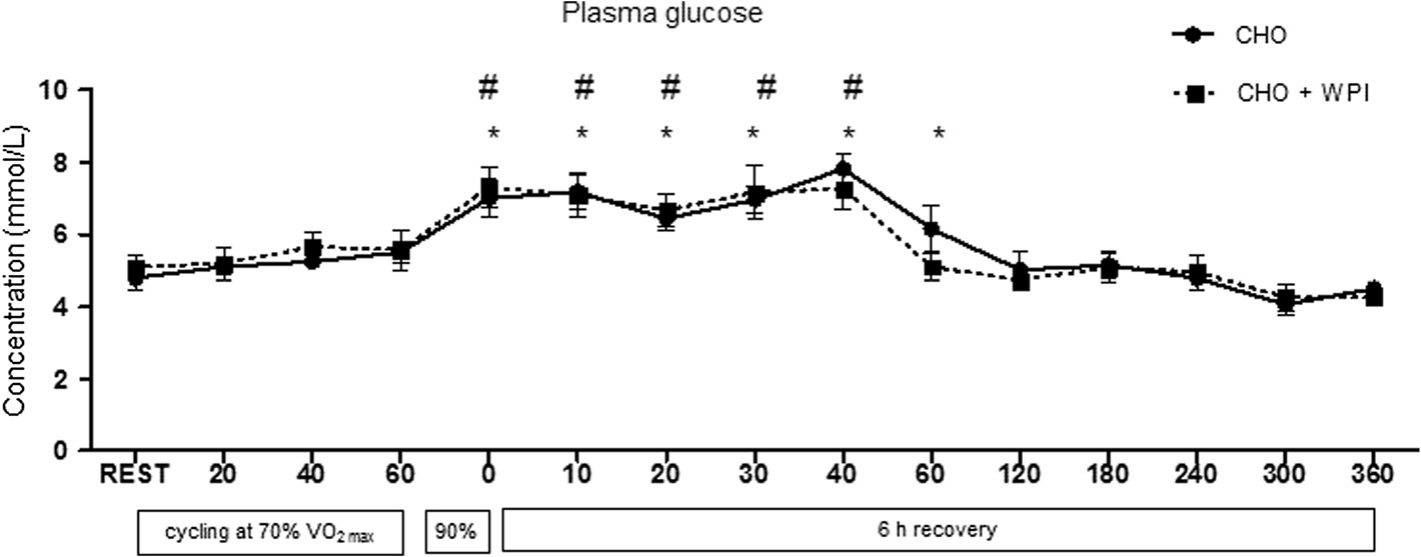
**Plasma glucose concentration for carbohydrate (CHO) and carbohydrate and whey protein isolates (CHO + WPI) trials. **The exercise trial day consisted of 60 min cycling at 70% VO_2 max_, with blood samples taken at rest and every 20 min (rest, 20, 40, 60). This was followed by time to fatigue at 90% VO_2 max _and blood was taken on completion of this effort (0). The 6 h recovery consisted of blood taken regularly for the first h (10, 20, 30, 40, 60) and every 60 min after that (120, 180, 240, 300, 360). Both CHO and CHO + WPI trials were significantly increased on completion of cycling at 90% VO_2__max _and remained elevated compared to rest until 40 min during recovery in the CHO + WPI trial (# P < 0.05). Whilst the CHO group remained elevated compared to rest until 60 min during recovery (* P < 0.05). Values are means ± SEM (n = 6).

**Figure 2:**
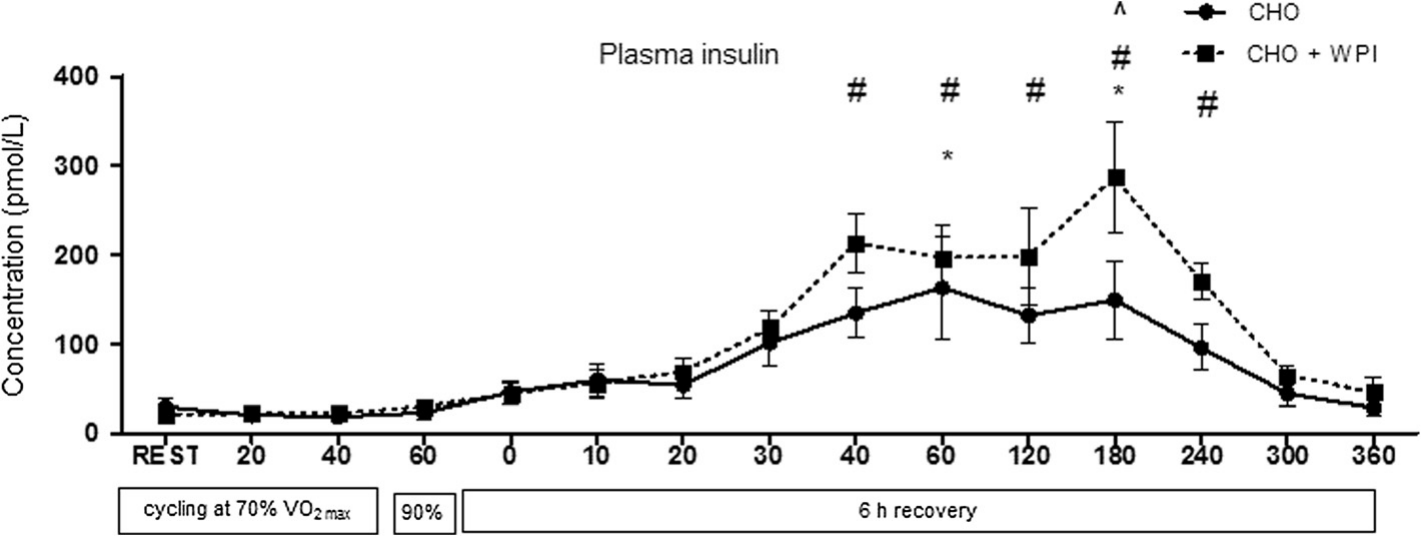
**Plasma insulin concentration for carbohydrate (CHO) and carbohydrate and whey protein isolates (CHO + WPI) trials. **The exercise trial day consisted of 60 min cycling at 70% VO_2 max_, with blood samples taken at rest and every 20 min (rest, 20, 40, 60). This was followed by time to fatigue at 90% VO_2 max _and blood was taken on completion of this effort (0). The 6 h recovery consisted of blood taken regularly for the first h (10, 20, 30, 40, 60) and every 60 min after that (120, 180, 240, 300, 360). Both trials, CHO (* P < 0.05) and CHO + WPI (# P < 0.05), were significantly elevated compared to rest, with CHO + WPI significantly higher than CHO at 180 min (^ P < 0.05) during the recovery period, before returning to resting levels at 240 min. Values are means ± SEM (n = 6).

Time to fatigue was not significantly different between CHO (11:14 ± 1:05 min) and CHO + WPI (10:05 ± 1:30 min). Plasma glucose concentration is presented in Figure 1. For both CHO and CHO + WPI groups, plasma glucose was significantly increased during cycling at 90% VO_2_ _max_ and remained elevated compared to rest until 40 min during recovery, with the CHO group remaining elevated until 60 min during recovery. No differences in plasma glucose were detected between the trials at any time point. Plasma insulin concentration (Figure 2) for the CHO trial increased compared to rest, from 40 min to 180 min during recovery (P < 0.05). The CHO + WPI trial increased compared to rest, from 30 min to 180 min during recovery (P < 0.05). The CHO + WPI trial had significantly elevated insulin levels at 180 min during the recovery period (P < 0.05) compared to CHO trial.

**Figure 3:**
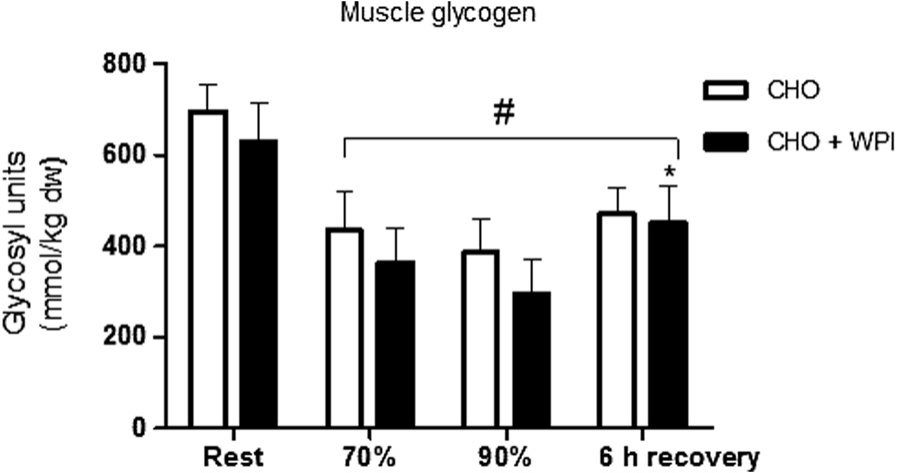
**Muscle glycogen concentration following the 16 day dietary intervention and exercise trial day, which consisted of a resting (rest) muscle biopsy, another following 60 min cycling at 70% VO**_**2 max **_**(70%)**_**, **_**time to fatigue at 90% VO**_**2 max **_**(90%) and at the end of 6 h recovery (6 h recovery).** Carbohydrate (CHO) and carbohydrate and whey protein isolates (CHO + WPI) trial were similar at rest. All time points following exercise were lower than rest in both trials (# P < 0.05). CHO + WPI trial was increased from 90% VO_2 max _to end of 6 h recovery (* P < 0.05). Values are means ± SEM (n = 6).

**Figure 4:**
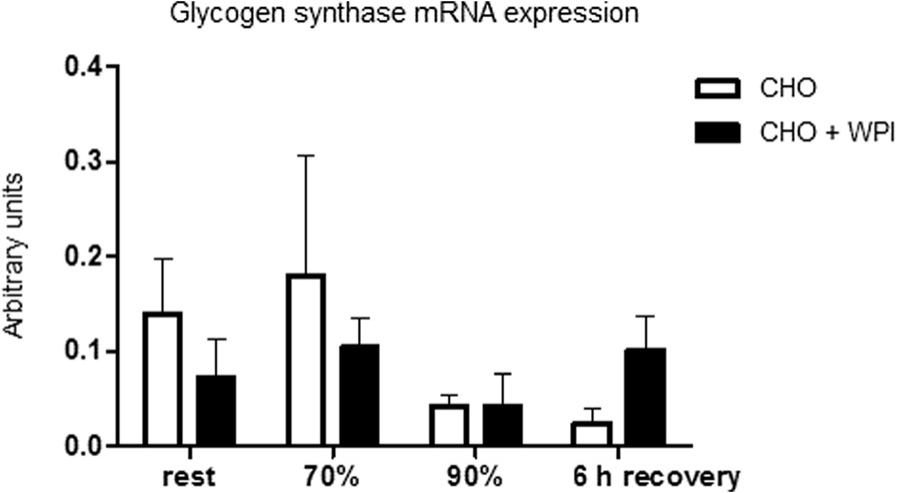
**Glycogen synthase mRNA expression for the carbohydrate (CHO) and carbohydrate and whey protein isolates (CHO + WPI) trials. **No differences were observed. Values are means ± SEM (n = 6).

Muscle glycogen content (Figure 3) was similar for CHO and CHO + WPI trials at rest. Following exercise and 6 h recovery period both trials were lower than rest (P < 0.05). The CHO + WPI trial was significantly increased from the end of cycling at 90% VO_2_ _max_ to the end of 6 h recovery, whereas the CHO trial did not show this increase. This occurred with no difference in mRNA expression of glycogen synthase between CHO and CHO + WPI trials (Figure 4).

**Figure 5:**
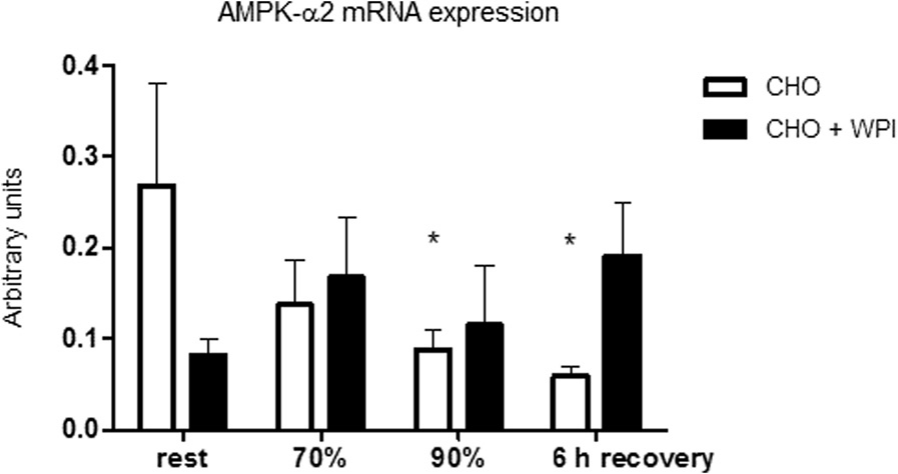
**AMPK-α2 mRNA expression for carbohydrate (CHO) and carbohydrate and whey protein isolates (CHO + WPI) trials. **CHO group is significantly different from rest to 90% and rest to end recovery (* P < 0.05). Values are mean ± SEM (n = 6).

**Figure 6:**
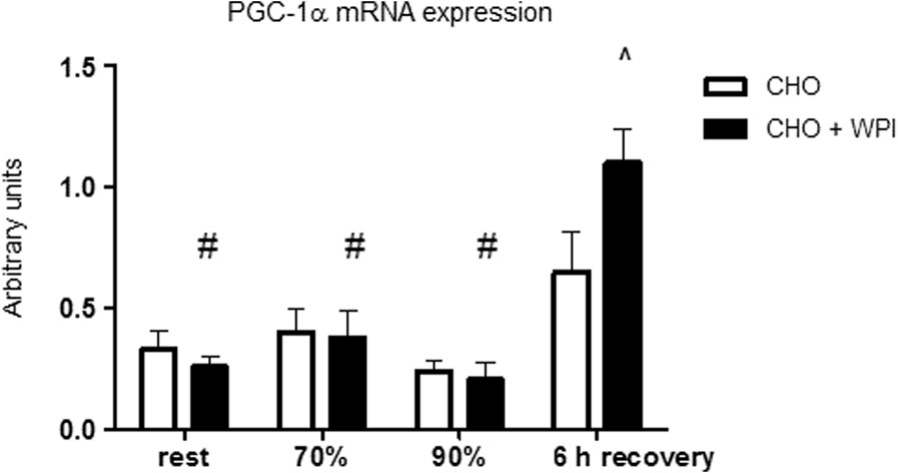
**PGC-1α mRNA expression for carbohydrate (CHO) and carbohydrate and whey protein isolate trials (CHO + WPI) following 16 day dietary intervention and exercise trial. **Muscle biopsies were taken at rest, another following 60 min cycling at 70% VO_2 max _(70%)_, _time to fatigue at 90% VO_2 max _(90%) and at the end of 6 h recovery (6 h recovery). CHO + WPI trial was significantly lower at rest, following cycling at 70% and 90% VO_2_ _max_, compared to 6 h recovery (# P < 0.05). After 6 h of recovery the CHO + WPI trial was significantly increased compared to CHO trial (^P < 0.05). Values are mean ± SEM (n = 6).

AMPK-α2 mRNA expression (Figure 5) was similar for CHO and CHO + WPI trials. Following cycling at 90% VO_2__max_ and end of 6 h recovery, the CHO trial was lower compared to rest (P < 0.05). PGC-1α mRNA expression (Figure 6) was significantly higher at the end of 6 h recovery compared to all other time points in the CHO + WPI trial (P < 0.05). Following 6 h recovery the CHO + WPI trial was significantly higher (P < 0.05) compared to the isocaloric carbohydrate matched CHO trial.

## Discussion

Protein is considered a key nutritional component for athletic success, however there appears to be a lack of information regarding the effect of combined CHO and protein supplementation on exercise adaptations during recovery. This study compared 2 weeks co-ingestion of whey protein isolates supplementation combined with a high carbohydrate diet with an iso-caloric carbohydrate matched diet in endurance athletes. Protein supplementation with adequate carbohydrate availability, included in a regular training program, did not influence intense aerobic cycling performance or pre- and post-exercise muscle glycogen levels. However, increases in plasma insulin and muscle PGC-1α mRNA expression with CHO + WPI supplementation compared to CHO alone indicates a potential for improved adaptations to training following supplementation.

Resting muscle glycogen levels were comparable with previously published carbohydrate loading protocols [25]. Supplementation with whey protein isolates does not further increase resting muscle glycogen levels when adequate CHO (8 g ^.^ kg^-1.^ bw/day) is consumed on a daily basis, followed by CHO loading prior to competition. However, glycogen resynthesis at the end of 6 h recovery was enhanced for the CHO + WPI trial and not the CHO trial. Earlier studies have shown co-ingestion of whey proteins with carbohydrate consumed during exercise and recovery period to augment muscle glycogen synthesis during the recovery period [26-28]. These studies used suboptimal levels of carbohydrate (< 0.8 g ^.^ kg^-1.^ bw/h) ingestion required for maximal glycogen synthesis rates during recovery, suggesting co-ingestion of CHO + WPI may only be beneficial for muscle glycogen resynthesis when insufficient CHO is consumed. However, the current study has also shown benefits of the addition of whey protein isolates even when optimal CHO is ingested.

Jentjens et al. [21] found co-ingestion of an amino acid mixture in combination with a large carbohydrate intake (1.2 g ^.^ kg^-1.^ bw/h) during recovery accentuates plasma insulin concentrations. The current study demonstrated increased insulin at 180 min of recovery following ingestion of the CHO + WPI sports beverage and a sustained elevation of insulin levels over a longer time. Whey protein isolates are insulinotrophic (the ability to stimulate the production of insulin) compared to caseins and other proteins of vegetable origin [29,30]. Whey protein isolates have been shown to induce an insulin response independent of carbohydrate co-ingestion [31].

Previous studies have suggested increased insulin levels to be one of the main mechanisms to increase muscle glycogen levels, via stimulation of glucose transporters in the muscle to increase glucose uptake along with the action of glycogen synthase [28,32]. Glycogen synthase mRNA expression was not increased in this study, indicative of a lack of stimulus for enhanced glycogen synthesis. However, the increased plasma insulin during recovery in the CHO + WPI trial may explain the enhanced recovery of muscle glycogen observed in the current study. The earlier reduction in plasma glucose concentration in the CHO + WPI trial (after 40 min) compared to CHO alone (after 60 min) supports this observation.

Insulin may also play a role in enhancing net protein balance by attenuating protein degradation [33]. Morrison et al. [34] examined the effect of endurance exercise and nutrition (CHO, protein and CHO + protein) on the signal transduction pathways involved in mRNA translation; the mammalian target of rapamycin (mTOR) and three of its dependent signalling proteins: ribosomal protein s6 kinase- 1 (p70^s6k^), ribosomal protein S6 (rps6) and elongation initiation factor 4E binding protein-1 (4E-BP1). The CHO + protein group demonstrated increased plasma insulin and phosphorylated states of 4E-BP1 and rpS6 at 30 min post exercise, compared to the CHO and protein alone groups. mTOR is also involved in the activation of mitochondrial biogenesis [35]. These observations are in agreement with the current study which demonstrated an increased insulin response in the CHO + WPI trial, which may have played a role in the increased PGC-1α mRNA expression observed.

Mitochondrial biogenesis is a well-established adaptation associated with endurance-type exercise [36], with PGC-1α and AMPK important regulators of this process in skeletal muscle [36,37]. Changes in cellular energy status activate AMPK, which in turn phosphorylates PGC-1α [36,38]. AMPK-α2 mRNA expression was decreased compared to rest in the CHO trial after cycling at 90% VO_2_ max and 6 h recovery, although this was not different to the CHO + WPI trial.

PGC-1α binds and co-activates a number of transcription factors from both the nuclear and mitochondrial genomes [36,39]. A single bout of physical activity has been shown to increase PGC-1α mRNA in humans [40,41]. The results from the current study demonstrated co-ingestion of CHO + WPI elevated PGC-1α mRNA expression compared to CHO at the end of the 6 h recovery period. This result may have important implications for consuming CHO + WPI with an endurance training program and enhancing muscle adaptations to training load. Numerous studies have investigated the effects of co-ingestion of carbohydrate and proteins during and after endurance-type exercise on protein synthesis rates and whole body protein balance [42,43]. However, these studies do not explore co-ingestion of CHO and proteins on signalling pathways involved in protein synthesis, in particular mitochondrial biogenesis signalling.

Breen et al. [44] investigated mitochondrial and myofibrillar muscle protein synthesis when carbohydrate or carbohydrate plus protein beverages were ingested following prolonged endurance cycling. This study found ingestion of carbohydrate plus protein increased myofibrillar but not mitochondrial muscle protein synthesis. This is in contrast to the current study, in which PGC-1α mRNA increased with CHO + WPI compared to CHO alone. Aerobic exercise, such as the prolonged cycling performed in the study by Breen et al. [44], represents a stimulus that would elicit adaptations such as mitochondrial biogenesis and mitochondrial protein synthesis, in which PGC-1α is considered a master regulator. The current study investigated mRNA 6 hours post exercise, whereas Breen et al. [44] measured protein synthesis 4 hours post exercise. The latter time point may be too soon after exercise and consumption of CHO plus protein beverage, to see an increase in mitochondrial proteins [36]. It is important to note, the current study included 2 weeks of dietary control and supplementation prior to the exercise trial and the Breen et al. [44] study only supplemented post exercise. The CHO intake of the trained cyclist in the Breen et al. [44] study was 5 g · kg^-1^ body weight · d^-1^, this is below current recommendations for athletes [45], whereas the current study used 8 g · kg^-1^ body weight · d^-1^, which may have also resulted in the different observations in these studies.

## Conclusions

Our study demonstrated a 2-week dietary intervention of co-ingestion CHO + WPI, had positive effects on aspects of endurance adaptations at the end of 6 h recovery, following an exercise bout. Muscle glycogen levels were not further increased pre exercise, however with WPI supplementation; there was enhanced recovery from 90% VO_2_ _max_ cycling to end 6 h recovery. Plasma insulin levels were increased with CHO + WPI during the recovery phase. PGC-1α mRNA was increased at the end of 6 h recovery following ingestion of CHO + WPI. Co-ingestion of CHO + WPI therefore appears to play an important role in endurance training adaptations via increasing plasma insulin and PGC-1α mRNA expression during recovery which may lead to enhanced recovery, mitochondrial biogenesis and thus ultimately performance.

## Abbreviations

WPI: Whey protein isolates; CHO: Carbohydrate; PGC-1α: Peroxisome proliferator-activated receptor gamma coactivator-1 alpha; MPS: Muscle protein synthesis; W: Watts; HR: Heart rate; mRNA: Messenger RNA; AMPK-α2: Adenosine monophosphate-activated protein kinase-alpha 2; SEM: Standard error of the mean.

## Competing interests

This work has been supported in part by MG Nutritionals, Melbourne, Australia. The authors declare no other competing interest.

## Authors’ contributions

KH, CGS, EG, AH and AM developed the study design. KH was in charge of subject recruitment, data collection and management, statistical analysis. EG was responsible for carrying out mRNA expression analysis. CGS, AH and AM participated in data collection. All authors contributed to drafting of the manuscript. All authors have read and approved the final manuscript.

## References

[B1] Rodriguez N Vislocky L Gaine P Dietary protein, endurance exercise, and human skeletal-musvercle protein turn Curr Opin Clin Nutr 2007 10 40 45 10.1097/MCO.0b013e3280115e3b 17143053

[B2] Hawley J Tipton K Millard-Stafford M Promoting training adaptations through nutritional interventions J Sport Sci 2006 24 7 709 721 10.1080/02640410500482727 16766500

[B3] Ivy J Regulation of muscle glycogen repletion, muscle protein synthesis and repair following exercise J Sports Sci Med 2004 3 131 138 2004 PMC3905295 24482590

[B4] Ha E Zemel M Functional properties of whey, whey components, and essential amino acids: mechanisms underlying health benefits for acticve people J Nutr Biochem 2003 14 251 258 10.1016/S0955-2863(03)00030-5 12832028

[B5] Cox GR Clark SA Cox AJ Halson SL Hargreaves M Hawley JA Jeacocke N Snow RJ Yeo WK Burke LM Daily training with high carbohydrate availability increases exogenous carbohydrate oxidation during endurance cycling J Appl Physiol 2010 109 1 126 134 10.1152/japplphysiol.00950.2009 20466803

[B6] Rowlands D Thorp R Rossler K Graham D Rockell M Efect of protein-rich feeding on recovery after intense exercise Int J Sport Nutr Exerc Metab 2007 17 521 543 18156659 10.1123/ijsnem.17.6.521

[B7] Jentjens R Jeukendrup A Determinants of post exericse glycogen synthesis during short term recovery Sports Med 2003 33 2 117 144 10.2165/00007256-200333020-00004 12617691

[B8] Rauch H Gibson A Lambert E Noakes T A signalling role for muscle glycogen in the regulation of pace during prolonged exercise Brit J Sport Med 2005 39 34 38 10.1136/bjsm.2003.010645 PMC1725021 15618337

[B9] Westerblad H Bruton JD Katz A Skeletal muscle: energy metabolism, fiber types, fatigue and adaptability Exp Cell Res 2010 316 18 3093 3099 10.1016/j.yexcr.2010.05.019 20580710

[B10] Kerksick C Harvey T Stout J Campbell B Wilborn C Kreider R Kalman D Ziegenfuss T Lopez H Landis J Ivy JL Antonio J International Society of Sports Nutrition position stand: nutrient timing J Int Soc Sports Nutr 2008 5 17 10.1186/1550-2783-5-17 18834505 PMC2575187

[B11] Maughan R Nutritional status, metabolic responses to exercise and implications for performance Biochem Soc 2003 31 6 1267 1269 10.1042/BST0311267 14641040

[B12] Hawley JA Burke LM Phillips SM Spriet LL Nutritional modulation of training-induced skeletal muscle adaptations J Appl Physiol 2011 110 3 834 845 10.1152/japplphysiol.00949.2010 21030665

[B13] Smith T Montain S Anderson D Young A Plasma amino acid responses after consumption of beverages with varying protein type Int J Sport Nutr Exerc Metab 2009 19 1 17 19403950 10.1123/ijsnem.19.1.1

[B14] Tang JE Moore DR Kujbida GW Tarnopolsky MA Phillips SM Ingestion of whey hydrolysate, casein, or soy protein isolate: effects on mixed muscle protein synthesis at rest and following resistance exercise in young men J Appl Physiol 2009 107 3 987 992 10.1152/japplphysiol.00076.2009 19589961

[B15] Tipton K Wolfe R Protein and amino acids for athletes J Sport Sci 2004 22 65 79 10.1080/0264041031000140554 14971434

[B16] Wilkinson SB Phillips SM Atherton PJ Patel R Yarasheski KE Tarnopolsky MA Rennie MJ Differential effects of resistance and endurance exercise in the fed state on signalling molecule phosphorylation and protein synthesis in human muscle J Physiol 2008 586 Pt 15 3701 3717 18556367 10.1113/jphysiol.2008.153916 PMC2538832

[B17] Camera DM Edge J Short MJ Hawley JA Coffey VG Early time course of Akt phosphorylation after endurance and resistance exercise Med Sci Sports Exerc 2010 42 10 1843 1852 10.1249/MSS.0b013e3181d964e4 20195183

[B18] Walker D Dickinson J Timmerman K Drummond M Reidy P Fry C Gundermann D Rasmussen B Exercise, amino acids, and ageing in the control of human muscle protein synthesis Med Sci Sports Exerc 2011 published ahead of Print 10.1249/MSS.0b013e318223b037 PMC3289515 21606874

[B19] Cribb PJ Williams AD Carey MF Hayes A The effect of whey isolate and resistance training on strength, body composition, and plasma glutamine Int J Sport Nutr Exerc Metab 2006 16 5 494 509 17240782 10.1123/ijsnem.16.5.494

[B20] Hulmi JJ Lockwood CM Stout JR Effect of protein/essential amino acids and resistance training on skeletal muscle hypertrophy: A case for whey protein Nutr Metab (Lond) 2010 7 51 10.1186/1743-7075-7-51 20565767 PMC2901380

[B21] Jentjens RL van Loon LJ Mann CH Wagenmakers AJ Jeukendrup AE Addition of protein and amino acids to carbohydrates does not enhance postexercise muscle glycogen synthesis J Appl Physiol 2001 91 2 839 846 11457801 10.1152/jappl.2001.91.2.839

[B22] Evans WJ Phinney SD Young VR Suction applied to a muscle biopsy maximizes sample size Med Sci Sports Exerc 1982 14 1 101 102 7070249

[B23] Lowry O Passonneau J A flexible system of enzymatic analysis 1972 New York: Academic

[B24] Livak KJ Schmittgen TD Analysis of relative gene expression data using real-time quantitative PCR and the 2(−Delta Delta C(T)) Method Methods 2001 25 4 402 408 10.1006/meth.2001.1262 11846609

[B25] Madsen K Pedersen PK Rose P Richter EA Carbohydrate supercompensation and muscle glycogen utilization during exhaustive running in highly trained athletes Eur J Appl Physiol Occup Phys 1990 61 5–6 467 472 10.1007/BF00236069 2079068

[B26] Ivy J Goforth HJ Damon B McCauley T Parsons E Price T Early post exercise muscle glycogen recovery is enhanced with a carbohydrate-protein supplement J Appl Physiol 2002 93 1337 1344 12235033 10.1152/japplphysiol.00394.2002

[B27] Van Loon L Saris W Kruijshoop M Wagenmeakers A Maximising postexercise muscle glycogen synthesis: carbohydrate supplementation and the aplication of amino acid or protein hydrolysate mixtures Am J Clin Nutr 2000 72 106 111 10871568 10.1093/ajcn/72.1.106

[B28] Zawadzki K Yaspelkis B Ivy J Carbohydrate-protein complex increases the rate of muscle glycogen storage after exercise J Appl Physiol 1992 72 1854 1859 1601794 10.1152/jappl.1992.72.5.1854

[B29] Nilsson M Holst J Bjorck I Metabolic effect of amino acid mixtures and whey protein in healthy subjects: studis using glucose equivalent drinks Am J Clin Nutr 2007 85 996 1004 17413098 10.1093/ajcn/85.4.996

[B30] Power O Hallihan A Jakeman P Human insulinotropic response to oral ingestion of native and hydrolysed whey protein Amino Acids 2009 37 333 339 10.1007/s00726-008-0156-0 18679613

[B31] Claessens M Saris W Van Baak M Glucagon ad insulin responses after ingestion of different amounts of intact and hydrolysed proteins Brit J Nutr 2008 100 61 69 18167171 10.1017/S0007114507886314

[B32] Van Loon L Saris W Verhagen H Wagenmakers A PLasma insulin responses following the ingestion of different amino acid/protein carbohydrate mixtures Am J Clin Nutr 2000 72 96 105 10871567 10.1093/ajcn/72.1.96

[B33] Rowlands DS Thomson JS Timmons BW Raymond F Fuerholz A Mansourian R Zwahlen MC Metairon S Glover E Stellingwerff T Kussmann M Tarnopolsky MA Transcriptome and translational signaling following endurance exercise in trained skeletal muscle: impact of dietary protein Physiol Genomics 2011 43 17 1004 1020 10.1152/physiolgenomics.00073.2011 21730029

[B34] Morrison PJ Hara D Ding Z Ivy JL Adding protein to a carbohydrate supplement provided after endurance exercise enhances 4E-BP1 and RPS6 signaling in skeletal muscle J Appl Physiol 2008 104 4 1029 1036 10.1152/japplphysiol.01173.2007 18239077

[B35] Cunningham JT Rodgers JT Arlow DH Vazquez F Mootha VK Puigserver P mTOR controls mitochondrial oxidative function through a YY1-PGC-1alpha transcriptional complex Nature 2007 450 7170 736 740 10.1038/nature06322 18046414

[B36] Hood DA Mechanisms of exercise-induced mitochondrial biogenesis in skeletal muscle Appl Physiol Nutr Metab 2009 34 3 465 472 10.1139/H09-045 19448716

[B37] Lin J Handschin C Spiegelman BM Metabolic control through the PGC-1 family of transcription coactivators Cell Metab 2005 1 6 361 370 10.1016/j.cmet.2005.05.004 16054085

[B38] Irrcher I Adhihetty PJ Joseph AM Ljubicic V Hood DA Regulation of mitochondrial biogenesis in muscle by endurance exercise Sports Med 2003 33 11 783 793 10.2165/00007256-200333110-00001 12959619

[B39] Koulmann N Bigard AX Interaction between signalling pathways involved in skeletal muscle responses to endurance exercise Pflugers Arch 2006 452 2 125 139 10.1007/s00424-005-0030-9 16437222

[B40] Akimoto T Pohnert SC Li P Zhang M Gumbs C Rosenberg PB Williams RS Yan Z Exercise stimulates Pgc-1alpha transcription in skeletal muscle through activation of the p38 MAPK pathway J Biol Chem 2005 280 20 19587 19593 10.1074/jbc.M408862200 15767263

[B41] Baar K Wende AR Jones TE Marison M Nolte LA Chen M Kelly DP Holloszy JO Adaptations of skeletal muscle to exercise: rapid increase in the transcriptional coactivator PGC-1 FASEB J 2002 16 14 1879 1886 10.1096/fj.02-0367com 12468452

[B42] Koopman R Pannemans D Jeukendrup A Gijsen A Senden J Halliday D Saris W Van Loon L Wagenmakers A Combined ingestion of protein and carbohydrate improves protein balance Am J Physiol Endocrinol and Metab 2004 287 E712 E720 10.1152/ajpendo.00543.2003 15165999

[B43] Beelen M Zorenc A Pennings B Senden J Kuipers H Van Loon L Impact of protein coingestion on muscle protein synthesis during continuous endurance type exercise Am J Physiol Endocrinol and Metab 2011 300 E945 E954 10.1152/ajpendo.00446.2010 21364122

[B44] Breen L Philp A Witard OC Jackman SR Selby A Smith K Baar K Tipton KD The influence of carbohydrate-protein co-ingestion following endurance exercise on myofibrillar and mitochondrial protein synthesis J Physiol 2011 589 Pt 16 4011 4025 21746787 10.1113/jphysiol.2011.211888 PMC3179999

[B45] Rodriguez NR Di Marco NM Langley S American College of Sports Medicine position stand. Nutrition and athletic performance Med Sci Sports Exerc 2009 41 3 709 731 10.1249/MSS.0b013e31890eb86 19225360

